# Decreasing Shigellosis-related Deaths without *Shigella* spp.–specific Interventions, Asia

**DOI:** 10.3201/eid1611.090934

**Published:** 2010-11

**Authors:** Pradip Bardhan, A.S.G. Faruque, Aliya Naheed, David A. Sack

**Affiliations:** Author affiliations: International Centre for Diarrhoeal Disease Research, Bangladesh, Dhaka, Bangladesh (P. Bardhan, A.S.G. Faruque, A. Naheed);; Johns Hopkins University Bloomberg School of Public Health, Baltimore, Maryland, USA (D.A. Sack)

**Keywords:** Bacteria, enteric infections, Shigella, shigellosis, mortality, Asia, Bangladesh, measles vaccine, vitamin A, research

## Abstract

In 1999, a review of the literature for 1966–1997 suggested that ≈1.1 million persons die annually of shigellosis, including ≈880,000 in Asia. Our recent review of the literature for 1990–2009 indicates that ≈125 million shigellosis cases occur annually in Asia, of which ≈14,000 are fatal. This estimate for illnesses is similar to the earlier estimate, but the number of deaths is 98% lower; that is, the lower estimate of deaths is associated with markedly reduced case-fatality rates rather than fewer cases. *Shigella* spp.–related deaths decreased substantially during a period without *Shigella* spp.–specific interventions. We speculate that nonspecific interventions, e.g., measles vaccination, vitamin A supplementation, and improved nutrition, may have led to the reduced number of shigellosis-related deaths.

## Abstract 2

Despite a high number of cases, deaths have decreased 98% since the 1980s.

In 1999, Kotloff et al. reviewed the literature to estimate the global incidence of shigellosis. On the basis of studies published during 1966–1997, they estimated ≈1.1 million shigellosis-related deaths annually, resulting from ≈164.7 million cases. Of these, ≈163.2 million cases occurred in developing countries, ≈80% of which occurred in Asia (*1*). These high estimates of illness and death have increased interest in identifying interventions, including new vaccines, that might reduce these astonishing numbers (*2**–**5*).

Several changes have occurred that might have altered this incidence. Shigellosis might be increasing because of increasing populations in *Shigella* spp.–endemic areas; because of increasing resistance to antimicrobial drugs among shigellae, especially in *S. dysenteriae* type 1 (the Shiga bacillus) (*6**–**8*); or because of increasing rates of HIV infection and AIDS in many countries, which might be influencing shigellosis incidence. On the other hand, incidence might be decreasing because of improved nutrition in many countries, improved delivery of healthcare in some areas, and more widespread use of measles vaccine (*9**,**10*) and vitamin A supplementation (*11*), which might reduce the severity of intestinal infections. The availability of fluoroquinolones, often used without prescription, also might lead to changing treatment practices because families might use antimicrobial drugs earlier during diarrheal illness or for other illnesses (*12*).

Shigellosis incidence might also have changed because of the overall reduction in diarrhea-related deaths through case management, including rehydration therapy and proper feeding (*13*). Because shigellosis is not primarily a dehydrating condition, hydration is not critical for patients with dysentery. Nonetheless, the consistent use of oral rehydration therapy for diarrhea may reduce illness from the persistent effects of repeated episodes of diarrhea, which is common in developing counties.

Thus, at the request of the World Health Organization, we reviewed the literature for 1990–2009 to estimate the current incidence of shigellosis. The earlier study by Kotloff et al. attempted to extrapolate from data from developing countries; however, most of the data were from Asia. Because the epidemiology of shigellosis may differ in Africa, we restricted our review to studies in Asian populations.

## Materials and Methods

Our review comprised studies identified through Medline. The initial studies were identified by a computer search of the multilingual scientific literature published since 1990. Articles derived by using the keywords *Shigella*, dysentery, bacillary, and shigellosis were linked with a set of other articles obtained by using the keywords incidence, prevalence, public health, death rate, mortality, surveillance, burden, distribution, and permutations of the root word epidemiol-. We conducted searches for each Asian country, except Japan and Israel. The resulting cross-linked set contained 319 articles, which we culled to 164 articles that were relevant to the goal of the search. Additional sources were located through consultations with experts in the field, proceedings of expert meetings, and the ongoing Diarrheal Diseases Surveillance Programme of the International Centre for Diarrheal Disease Research, Bangladesh (ICDDR,B). To enable comparison over time, we adopted the methods of the previous review on shigellosis (*1*).

We created an algorithm to estimate the number of *Shigella* infections that occurred each year in Asia. In a preliminary step, the world’s population was divided into 4 age strata (0–11 months, 1–4 years, 5–14 years, and >15 years). Published rates of diarrhea for each of the 4 strata were used to estimate the diarrheal disease incidence. An increase in the severity of a patient’s illness influences the proportion of diarrheal episodes attributable to *Shigella* spp. This correlation can be presumed to strengthen as the proportion of *Shigella* infections increases because sampling progresses from cases of diarrhea detected by household surveillance to those among outpatients to persons admitted to hospital (*14*). Thus, we subdivided the total diarrheal disease incidence into these 3 settings: estimates of mild cases in persons who stayed at home; more severe cases needing care at a clinic but not hospitalization; and cases requiring hospitalization.

The total number of diarrhea cases attributable to shigellosis was calculated for the <1-year and 1–4-year age groups by multiplying the number of diarrhea cases in the 2 settings (community and treatment facilities) by the percentage of diarrhea cases from which *Shigella* spp. were isolated (Table 1). For older children and adults, we calculated total cases by multiplying the median percentages of diarrhea cases attributable to shigellosis in persons 5–14 years and >15 years of age by the number of diarrhea cases in these 2 age groups according to clinical setting (Table 2).

**Table 1:** Estimated annual number of diarrheal episodes in children 0–4 years of age, Asia, 1990–2009

Characteristic	Age group		
	0–11 m	1–4 y	0–4 y
Total population, × 1,000	78,533	282,719	361,252
No. diarrheal episodes/child/y (*15*)	3.2	2.3	
Total diarrheal episodes, × 1,000	251,306	650,252	901,559
At home (*14**,**16*)	221,651	597,583	819,234
At treatment facility (*14**,**16*)	29,655	52,670	82,325
In outpatient department	25,884	51,370	77,254
Hospitalized	3,771	1,300	5,071
Median caused by shigellosis, %			
At home*			4.4%
In treatment facility†	5.8	9.4	
Weighted mean caused by shigellosis, % (95% confidence interval)			5.1 (4.0–5.7)
No. shigellosis cases			
At home, × 1,000			39,669
In treatment facility, × 1,000	1,720	4,951	6,671
Total, × 1,000			46,340
95% confidence interval of weighted mean, × 1,000			44,924–57,316

* Technical Appendix Table 1 (. † Technical Appendix Table 2.

**Table 2:** *Shigella* spp.–associated diarrhea in older children and adults, Asia, 1990–2009

Characteristic	Age group, y	
	5–14	>15
Population (× 1,000)	742,911	2,833,857
Diarrhea episodes/person/y (*17*)*	0.65	0.50
Total diarrhea episodes, × 1,000	482,892	1,416,929
At home	473,234	1,388,590
Treatment facility (*1*)†	9,658	28,339
Median caused by *Shigella* spp., %		
At home*	4	4
At treatment facility†	11.6	10.7
Proportion caused by shigellosis (weighted mean), %		
At home*	4.6 (4.0–5.1)	
At treatment facility†	8.3 (7.7–9.0)	
Annual no. episodes of *Shigella* spp.–associated diarrhea, × 1,000		
At home	18,929	55,544
At treatment facility	1,120	3,032
Total no. episodes of *Shigella* spp.–associated diarrhea, × 1,000	20,049	58,576
95% confidence interval of weighted mean, × 1,000	19,673–24,898	57,726–73,057

* Technical Appendix Table 3. † Technical Appendix Table 4.

We adopted the estimates of Kosek et al. (*15*) to calculate the number of diarrhea episodes per person per year within countries in Asia (Table 1). These estimates were based on the review of 13 longitudinal studies of stable populations in 8 countries in Asia, where active surveillance was conducted during 1984–1995.

We estimated the proportion of diarrheal episodes in each stratum that can be attributed to shigellosis by analyzing only studies in which surveillance was conducted since 1990 and that used microbiologic confirmations to report the percentage of *Shigella* spp.–related diarrhea cases for the specified age group. An overall median percentage of shigellosis was then calculated for each stratum and multiplied by the total number of diarrheal cases in the stratum to derive the number of shigellosis cases in each stratum. In addition to the median, a weighted mean with 95% confidence intervals (CIs) was calculated for these analyses by using Freeman-Tukey transformed proportions. Weights used were equal to the inverse standard errors of these transformed proportions (*18**,**19*). The numbers of shigellosis cases were added to give an overall estimate of shigellosis-related illness. Case-fatality rates (CFRs) for persons hospitalized with *Shigella* infection at the ICDDR,B hospital were used to calculate age-specific rates of *Shigella* spp.–associated death. This hospital treats >100,000 diarrhea patients annually and is the same hospital used for CFRs in the earlier study.

Illness was expressed as episodes of diarrhea per person-year from which shigellae were recovered. Studies were included in the death estimates if deaths caused by *Shigella* spp. could be ascertained through active surveillance. The review comprised prospective and retrospective studies but not studies based on vital statistics only. Death was considered to have been caused by diarrhea only if diarrhea was listed as the primary cause.

## Results

Approximately 3,938,020,000 persons resided in Asia during 2005. This estimate included 78,533,000 infants <1 year of age and 361,252,000 children 1–4 years of age (*20**,**21*).

### Shigellosis Incidence

The median frequency of *Shigella* spp. isolation from diarrheal cases in the community in children 0–4 years of age was 4.4% (range 3.1%–13.4%; weighted mean 5.1%, 95% CI 4.4%–5.7%). Because only 1 study broke this rate down into the <1-year and 1–4-year ranges, the median of the 2 values for the combined range was calculated. The median frequencies of *Shigella* spp. isolation rates from persons with diarrhea reporting to the treatment facilities were 5.8% (range 2.4%–9.3%) among children <1 year of age and 9.4% (range 2.4%–23.5%) among children 1–4 years of age. The weighted mean of the combined group was 6.6% (95% CI 6.0%–7.2%). Details of these studies are found in Technical Appendix Tables 1, 2).

Approximately 39,669,000 (weighted mean 45,980,000, 95% CI 39,669,000–51,389,000) shigellosis cases occurred in children <5 years of age in the community and 6,671,000 (weighted mean 5,433,000, 95% CI 5,256,000–5,927,000) in treatment facilities, totaling 46,717,000 (95% CI 44,924,000–57,316,000) cases among Asian children <5 years of age annually. The proportions of cases with shigellosis are detailed in Technical Appendix Tables 1, 2.

The median percentage of diarrhea in the community was 4.0% (range 1.6%–13.5%; weighted mean 4.6%, 95% CI 4.0%–5.1%). The median percentages for patients treated at facilities were ≈11.6% (range 4.7%–17.3%) and ≈10.7% (range 4.1%–27%) respectively (weighted mean of the combined groups 8.3%, 95% CI 7.7–9.0%). (The proportions of shigellosis cases are detailed in Technical Appendix Table 3, 4.) *Shigella* infections among children 5–14 years of age and persons >15 years of age were ≈20,049,000 (95% CI 19,673,000–24,898,000) and ≈58,576,000 (95% CI 57,726,000–73,057,000), respectively.

**Table 3:** Case-fatality rates for hospitalized patients with *Shigella* infections, Asia, 1990–2009

Country	Area	Year	Case-fatality rate by age group		
			0–11 mo	1–4 y	>5 y
Bangladesh*	Urban/rural	1990–1999	2.73	1.42	0.33
Bangladesh†	Urban	2000–2008	1.26	0	0
Bangladesh‡	Rural	2000–2008	0.51	0.02	0
People’s Republic of China (*22*)	Rural	2002	0	0	0
Thailand (*22*)	Rural	2000–2003	0	0	0
Indonesia (*22*)	Rural	2001–2003	0	0	0
Vietnam (*22*)	Urban/rural	2001–2003	0	0	0
Pakistan (*22*)	Rural	2002–2003	0	0	0
Bangladesh (*22*)	Periurban	2002–2004	0	0	0
Median			0.89	0.02	0
Weighted mean, % (95% confidence interval)			0.80 (0.59–1.03)	0.1 (0.02–0.25)	

*International Centre for Diarrhoeal Disease Research, Bangladesh (ICDDRB,B) hospital surveillance, 1990–1999. †ICDDR,B hospital surveillance (urban), 2000–2008. ‡ICDDR,B hospital surveillance (rural), 2000–2008.

**Table 4:** Estimated annual number of deaths and case-fatality rates for hospitalized persons with *Shigella* infection, Asia, 1990–2009*

Characteristic	Age group

0–11 mo	1–4 y
No. persons with diarrhea, × 1,000†	3,771	1,300
No. persons with *Shigella* infection, × 1,000 (% total persons with diarrhea)†	219 (5.8)	122 (9.4)
No. *Shigella* spp.–related deaths (95% CI)	1,949 (1,292–2256)	24 (24–305)
Case fatality rate, %‡	0.89	0.01
Corrected no. *Shigella* spp.–related deaths, × 1,000 (95% CI)§ (*23*,*24*)	13,643 (9,044–15,792)	168 (168–2,135)
Total no. *Shigella* spp.–related deaths (95% CI)	13,811 (9,212–17,927)	

*CI, confidence interval. †Table 1. ‡Table 3. §Corrected for out-of-hospital deaths.

We combined the number of shigellosis episodes in all age groups. The total annual number of shigellosis cases in Asia was ≈125 million (95% CI 122 million–155 million).

### Shigellosis-associated Deaths

Median CFRs for hospitalized shigellosis patients <1 year and 1–4 years of age and patients >5 years of age were 0.89%, 0.01%, and 0 respectively (Table 3), according to data from the ICDDR,B hospital surveillance program during 1990–2007. The weighted means for patients <1 and 1–4 years of age were 0.8% (95% CI 0.5%–1.0%) and 0.1% (95% CI 0.02%–0.25%), respectively. No deaths were reported from large studies in other countries in Asia. By using median CFRs from Bangladesh for these age groups, we determined that ≈1,960 shigellosis deaths occurred in Asia among hospitalized patients annually (Table 3). By using the 95% CIs, we estimated that the number of deaths ranged from 1,347 to 2,595.

A study from Bangladesh found that only 17.8% of shigellosis-related deaths occurred in treatment facilities; another study from the Gambia reported that only 12% of deaths associated with *Shigella* infection among children occurred in a health center (*23**,**24*). Thus, the true number of shigellosis-associated deaths may be 6–8× higher than deaths recorded in the hospital records. Hence, the estimates of the in-hospital shigellosis-associated deaths were multiplied by a factor of 7 in all age groups to correct for out-of-hospital mortality. This increased the number of deaths in all age groups to ≈13,720 shigellosis-related deaths across all the age groups per year in Asia (Table 4).

## Discussion

Our review calculated that ≈125 million cases of endemic shigellosis occur annually in Asia, of which ≈14,000 (0.011%) cases result in death. Children <5 years of age are at highest risk for *Shigella* spp.–related illness and death. Although this estimate suggests that shigellosis incidence is substantial and similar to the earlier estimate, the updated death estimate is 98% lower than the estimate by Kotloff et al. (*1*) that used data primarily from the 1980s. Assuming that the population of Asia is ≈80% of the total population of the developing countries, ≈130 million *Shigella* infections and ≈880,000 deaths occurred in Asia according to the earlier estimate.

With such a large difference in estimated incidences, one estimate may be more accurate than the other. Alternatively, *Shigella* spp.–related deaths may have decreased substantially since the 1980s, even in the absence of specific interventions against shigellosis. We believe the latter explanation best explains the large difference in estimates of deaths.

The major variable that was lower in our calculations was the CFR for hospitalized patients, especially children. In the earlier estimate, a CFR of 11% was used from the ICDDR,B hospital (*24*). Recent data from the same hospital indicate the rate is now ≈0.01% overall and only 0.89% for the youngest age group. A recent estimate from Africa found a CFR of <1% during an outbreak associated with *S. dysenteriae*, suggesting that this low CFR may not be limited to Asia (*25*).

The decrease in CFRs could be explained by >1 factor. Case management might have improved, strains might be less virulent, or children might be healthier when they become infected and therefore have less severe complications. Case management in the hospital is unlikely to have changed substantially, and in fact the increasing resistance of current strains to antimicrobial drugs makes case management more difficult. Case management in the home may have changed, however, because antimicrobial drugs are widely available, and families may purchase effective antimicrobial drugs, e.g., ciprofloxacin, and begin treatment earlier in the course of the illness (*12*). Virulence of infecting strains could be lower; infections with S*. dysenteriae* type 1 are unusual. During past epidemics with this serotype, however, the CFR for *S. flexneri* was as high as it was with *S. dysenteriae* type 1 (*24*). Thus, virulence is unlikely to explain the decrease in the number of deaths.

Improved health of children who become infected appears to best explain the decreased CFR. Nutritional status of children in Bangladesh has continued to improve slowly (*26*). Perhaps more essential is the high proportion receiving measles vaccine and vitamin A (*27*). Anecdotally, in children dying of shigellosis during the 1980s, postmeasles dysentery was often diagnosed, and measles increased the severity of diarrhea, including shigellosis (*9**,**10*). Measles with dysentery is rarely seen now in Bangladesh.

Our review has some limitations. Although we reviewed all available published data on shigellosis in Asia since 1990, few sites conduct active surveillance for this infection, and only one estimates CFRs. With this large population, extrapolating accurately to the entire continent might not be possible. Nevertheless, the same methods were used in this and the earlier review. The large multicenter study on shigellosis in Asia did not record any deaths, suggesting that fatalities from shigellosis are not common (*22*).

Second, the review included only Asia, and the situation in Africa is possibly (even likely) different (*28*). The higher rates of HIV infection and AIDS and malaria, different nutritional deficiencies, different rates of measles vaccination, and different health systems and civil disturbance might suggest higher *Shigella* spp.–related deaths in Africa. Unfortunately, until recently, no long-term surveillance for diarrhea has existed in Africa on which to base estimates.

Third, the data in the review were based on microbiologic diagnosis of *Shigella* infections. Although isolation of *Shigella* spp. from fecal samples is the most specific diagnostic test for shigellosis, the culture method has limited sensitivity because of the relatively fastidious nature of the organism. Adoption of improved specimen transport methods and newer and more sensitive molecular laboratory diagnostic methods (e.g., PCR) reportedly having high sensitivities may detect more infections (*29*) but is unlikely to alter the death estimates.

The remarkable 98% decrease in deaths from shigellosis in the absence of a *Shigella* spp.–specific intervention suggests that other nonspecific interventions have helped to lower *Shigella* spp.–specific deaths. These, we believe, include measles vaccine, vitamin A supplements, and overall improvement in nutrition. Although the ready availability of antimicrobial drugs encourages the development of antibiotic drug resistance because of frequent abuse, we cannot rule out the possibility that the rapid availability of these antibiotics (especially fluoroquinolones) also have benefited children with dysentery who may be receiving treatment more quickly than they previously did.

The findings from our review may provide lessons regarding other infectious diseases. Approximately 50% of deaths among children <5 years of age have malnutrition as an underlying cause (*30*). Also, malnutrition and infection are clearly related, with one leading to the other. By reducing rates of other common infections, e.g., measles, and improving the nutritional status, including micronutrient nutrition, of children, diseases from other infections, such as *Shigella* spp., may decrease.

The 4 species and numerous serotypes of *Shigella* spp. are a challenge for vaccine developers, but shigellosis remains high, and increasing resistance to antibiotic drugs continues to make treatment difficult. An effective *Shigella* spp. vaccine may have substantial benefits, but our study suggests that *Shigella* spp.–related deaths can be, and have been, substantially reduced with currently available interventions and that such interventions do not need to be *Shigella* spp. specific.

## Supplementary Material

Technical Appendix Proportion and total number of diarrheal episodes data tables.
