# Supplementary material for: Decreasing Shigellosis-related Deaths without Shigella spp.–specific Interventions, Asia
Source: Emerg Infect Dis. 2010 Nov;16(11):1718–23. doi: 10.3201/eid1611.090934 (PMC3294502; doi:10.3201/eid1611.090934)
Supplement: Technical Appendix — Proportion and total number of diarrheal episodes data tables. [file 09-0934-Techapp.pdf]

Article DOI: 10.3201/eid1611.090934

Decreasing Shigellosis-related Deaths without *Shigella* spp.–specific Interventions, Asia

Technical Appendix

Technical Appendix Table 1. Proportion and total number of diarrheal episodes in which *Shigella* was detected in children 0–4 years in community-based studies, Asia, 1990–2009

Country (reference)	Period	Setting	No. of diarrhea episodes	0–11 mo	1–4 y	0–4 y
Vietnam (*1*)	1998–1999	Rural	2,160	5.4%	6.7%	6.2%
India (*2*)	1998–1999	Rural	364			4.4%
China (*3*)	2002	Rural	1,851			3.1%
Thailand (*3*)	2000–2003	Rural	1,618			4.3%
Indonesia (*3*)	2001–2003	Rural	10,998			5.3%
Vietnam (*3*)	2001–2003	Rural	4,820			4.3%
Pakistan (*3*)	2002–2003	Rural	8,084			3.6%
Bangladesh (*3*)	2002–2004	Periurban	2,579			13.4%
		Median, %		4.4		
		Weighted mean (95% confidence interval), %		5.1 (4.4–5.7)		
Total estimated community-based cases: 39,669,000 (4.4% of 901,559,000)*						

*From Table 1.

Technical Appendix Table 2. Proportion and total number of diarrheal episodes (in %) in which *Shigella* was detected in children 0–4 years in treatment facility–based studies, Asia, 1990–2009

Country (reference)	Period	Setting	No. diarrhea cases	Proportion *Shigella*, %	
				0–11 mo	1–4 y
Bangladesh (*4*)	1993–1994	Urban	814	5.9	13.9
Laos (*5*)	1994–1995	Urban	183	3.5	9.4
Thailand (*6*)	1995–1996	Urban	105		7.6
Saudi Arabia (*7*)	1995–1996	Urban	150	6.7	
Laos (*8*)	1996–1997	Urban	672	9.3	23.5
Yemen (*9*)	1998	Urban	561	6.4	7.2
India (*10*)	1995–2000	Urban	2,855		
Iran (*11*)	2001–2002	Urban/Rural	422	9.3	12.4
Indonesia (*12*)	2005–2008	Urban	12,670	←——- 2.4——→	
Bangladesh*	1990–1999	Urban/Rural	18,222	5.1	12.3
Bangladesh†	2000–2008	Urban	10,819	4.0	7.1
Bangladesh‡	2000–2008	Rural	8,186	5.1	14.9
		Median		5.8	9.4
		Weighted mean (95% confidence interval), %		6.6 (6.0–7.2)	

*International Centre for Diarrhoeal Disease Research, Bangladesh (ICDDRB,B) hospital surveillance system.
†ICDDRB hospital surveillance system (urban).
‡ICDDRB hospital surveillance system (rural).

Technical Appendix Table 3. Proportion of diarrheal episodes in which *Shigella* was detected in older children and adults in community-based studies, Asia, 1990–2009

Country (reference)	Period	Setting	Diarrhea episodes, no. (%)
China (*3*)	2002	Rural	8,253 (3.3)
Thailand (*3*)	2000–2003	Rural	4,918 (1.6)
Indonesia (*3*)	2001–2003	Rural	5,227 (11.8)
Vietnam (*3*)	2001–2003	Urban/Rural	5,438 (3.4)
Pakistan (*3*)	2002–2003	Rural	2,287 (4.6)
Bangladesh (*3*)	2002–2004	Periurban	885 (13.5)
Median			(4.0)
Weighted mean (95% confidence interval)			4.6 (4.0–5.1)

Technical Appendix Table 4. Proportion of diarrheal episodes in which *Shigella* was detected in older children and adults a treatment facility–based studies, Asia, 1990–2009

Country (reference)	Period	Setting	No. diarrhea cases	Proportion *Shigella* spp. Positive, %	
				5–14 y	≥15 y
Laos (*5*)	1994–95	Urban	80	12.5	
Laos (*8*)	1996–97	Urban	106		10.6
Pakistan (*13*)	1997–99	Rural	264	<——13——>	
Iran (*11*)	2001–02	Urban/Rural	312	17.3	27
Bangladesh*	1990–99	Urban/Rural	15,136	<——10.7——>	
Bangladesh†	2000–08	Urban	8,987	4.7	4.1
Bangladesh‡	2000–08	Rural	5,324	8.6	9.8
Median				11.6	10.7
Weighted mean (95% confidence interval)				8.3 (7.7–9.0)	

*International Centre for Diarrhoeal Disease Research, Bangladesh (ICDDR,B) hospital surveillance system.
†ICDDR,B hospital surveillance system (urban).
‡ICDDR,B hospital surveillance system (rural).

References

1. Isenbarger DW, Hien BT, Ha HT, Ha TT, Bodhidatta L, Pang LW, et al. Prospective study of the incidence of diarrhoea and prevalence of bacterial pathogens in a cohort of Vietnamese children along the Red River. Epidemiol Infect. 2001;127:229–36. PubMed DOI: 10.1017/S0950268801005933

2. Gupta DN, Sircar BK, Sengupta PG, Ghosh S, Banu MK, Mondal SK, et al. Epidemiological and clinical profiles of acute invasive diarrhoea with special reference to mucoid episodes: a rural community-based longitudinal study. Trans R Soc Trop Med Hyg. 1996;90:544–7. PubMed DOI: 10.1016/S0035-9203(96)90315-6

3. von Seidlein L, Kim DR, Ali M, Lee H, Wang X, Thiem VD, et al. A multicentre study of *Shigella* diarrhoea in six Asian countries: disease burden, clinical manifestations, and microbiology. PLoS Med. 2006;3:e353. PubMed DOI: 10.1371/journal.pmed.0030353

4. Albert MJ, Faruque AS, Faruque SM, Sack RB, Mahalanabis D. Case-control study of enteropathogens associated with childhood diarrhea in Dhaka, Bangladesh. J Clin Microbiol. 1999;37:3458–64. PubMed

5. Phetsouvanh R, Midorikawa Y, Nakamura S. The seasonal variation in the microbial agents implicated in the etiology of diarrheal diseases among children in Lao People's Democratic Republic. Southeast Asian J Trop Med Public Health. 1999;30:319–23. PubMed

6. Suwatano O. Acute diarrhea in under five-year-old children admitted to King Mongkut Prachomklao Hospital, Phetchaburi province. J Med Assoc Thai. 1997;80:26–33. PubMed

7. el-Sheikh SM, el-Assouli SM. Prevalence of viral, bacterial and parasitic enteropathogens among young children with acute diarrhoea in Jeddah, Saudi Arabia. J Health Popul Nutr. 2001;19:25–30. PubMed

8. Yamashiro T, Nakasone N, Higa N, Iwanaga M, Insisiengmay S, Phounane T, et al. Etiological study of diarrheal patients in Vientiane, Lao People's Democratic Republic. J Clin Microbiol. 1998;36:2195–9. PubMed

9. Banajeh SM, Ba-Oum NH, Al-Sanabani RM. Bacterial aetiology and anti-microbial resistance of childhood diarrhoea in Yemen. J Trop Pediatr. 2001;47:301–3. PubMed DOI: 10.1093/tropej/47.5.301

10. Dutta S, Rajendran K, Roy S, Chatterjee A, Dutta P, Nair GB, et al. Shifting serotypes, plasmid profile analysis and antimicrobial resistance pattern of shigellae strains isolated from Kolkata, India during 1995–2000. Epidemiol Infect. 2002;129:235–43. PubMed DOI: 10.1017/S0950268802007240

11. MoezArdalan K, Zali MR, Dallal MM, Hemami MR, Salmanzadeh-Ahrabi S. Prevalence and pattern of antimicrobial resistance of *Shigella* species among patients with acute diarrhoea in Karaj, Tehran, Iran. J Health Popul Nutr. 2003;21:96–102. PubMed

12. Punjabi NH, Listiyaningsih E. Sedyaningsih, Agtini MD, Lay BW, Yogiara, et al. Identification and characterization of *Shigella* sp. as a cause of diarrhea among children in Indonesia. In: Proceedings of the Asian Conference on Diarrhoeal Diseases and Nutrition; 2009 May 25–27; Yogykarta, Indonesia.

13. Ahmed K, Shakoori FR, Shakoori AR. Aetiology of shigellosis in northern Pakistan. J Health Popul Nutr. 2003;21:32–9. PubMed
